# Supplementary figures and images for: Exploring the spatial association between the distribution of temperature and urban morphology with green view index
Source: PLoS One. 2024 May 14;19(5):e0301921. doi: 10.1371/journal.pone.0301921 (PMC11093354; doi:10.1371/journal.pone.0301921)

Appendix 1. Average monthly temperature difference in the four seasons from 2018 to 2021 at a cell size of 0.01゜*0.01゜


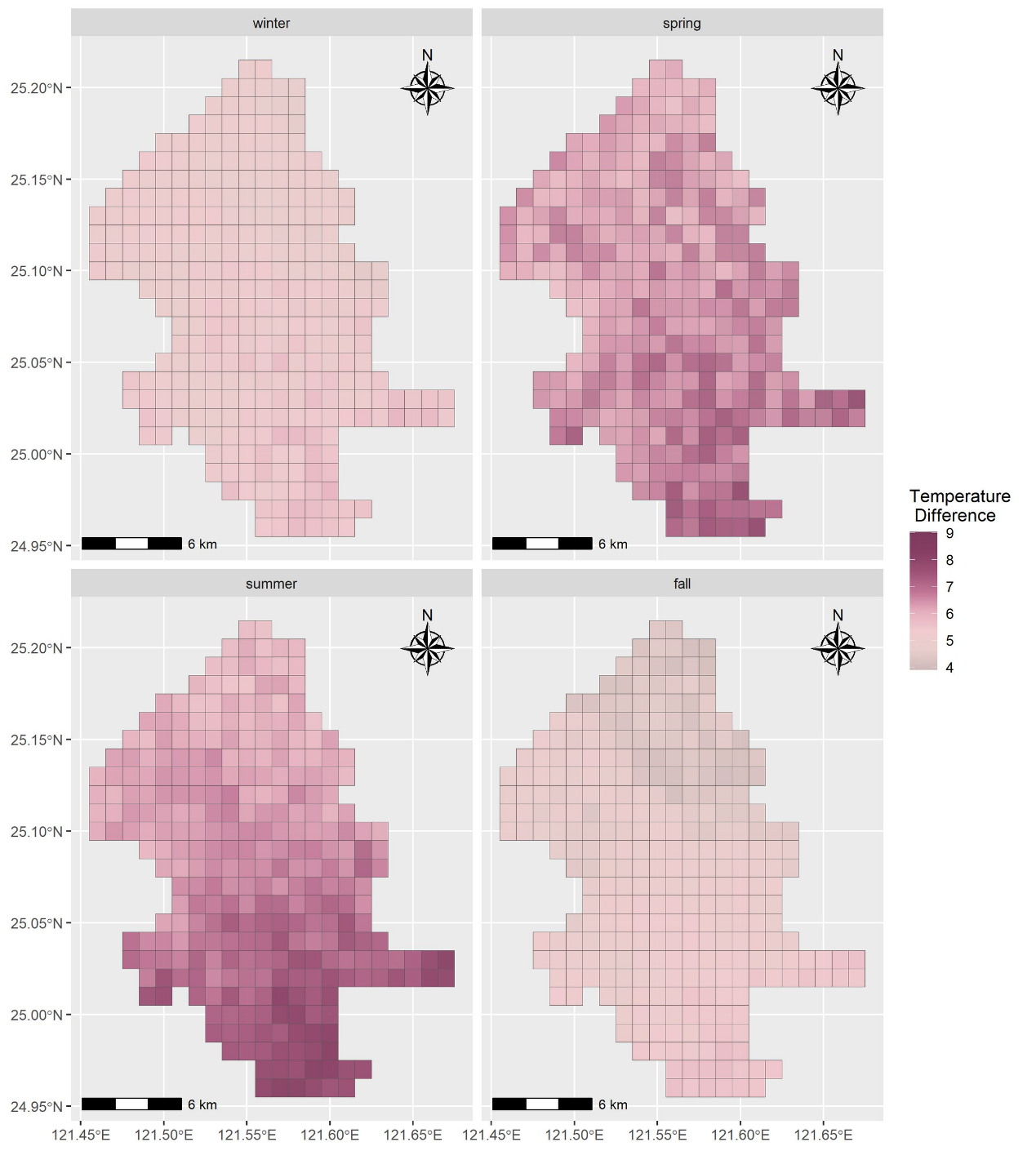

Supplement: S1 Appendix — (DOCX) [file pone.0301921.s001.docx]
