# Supplementary material for: Exploring the spatial association between the distribution of temperature and urban morphology with green view index
Source: PLoS One. 2024 May 14;19(5):e0301921. doi: 10.1371/journal.pone.0301921 (PMC11093354; doi:10.1371/journal.pone.0301921)
Supplement: S2 Appendix — (DOCX) [file pone.0301921.s002.docx]

Appendix 2. Results of the linear mixed model on the average monthly temperature difference

| Variables | Estimate | Std. Error | P-value | VIF |
| --- | --- | --- | --- | --- |
| Year | -0.099 | 0.014 | <0.001 | 1.000 |
| GVI2 | -0.005 | 0.002 | 0.002 | 1.159 |
| Season (ref=fall) |  |  |  | 1.045 |
| Spring | 1.616 | 0.05 | <0.001 |  |
| Summer | 1.644 | 0.093 | <0.001 |  |
| Winter | 0.216 | 0.058 | <0.001 |  |
| SVF | -0.006 | 0.002 | 0.002 | 1.056 |
| Land use: funeral | 0.015 | 0.005 | 0.005 | 1.009 |
| Adjusted R-squared: 71.8 %; AIC: 1826.8 | | | | |
