# Supplementary material for: Exploring the spatial association between the distribution of temperature and urban morphology with green view index
Source: PLoS One. 2024 May 14;19(5):e0301921. doi: 10.1371/journal.pone.0301921 (PMC11093354; doi:10.1371/journal.pone.0301921)
Supplement: S3 Appendix — (DOCX) [file pone.0301921.s003.docx]

Appendix 3. Results of the geographically weighted regression and simple linear regression on average monthly temperature differences

| Variables | Min. | Q1 | Median | Q3 | Max. | Coefficients^a^ |
| --- | --- | --- | --- | --- | --- | --- |
| Year | -0.351 | -0.161 | -0.077 | -0.098 | -0.019 | -0.099** |
| GVI2 | -0.027 | -0.008 | -0.001 | -0.001 | 0.005 | -0.005** |
| Season (ref=fall) |  |  |  |  |  |  |
| Spring | 1.087 | 1.386 | 1.552 | 1.534 | 1.659 | 1.628** |
| Summer | 0.907 | 1.449 | 1.576 | 1.695 | 2.078 | 1.613** |
| Winter | -0.273 | 0.109 | 0.223 | 0.215 | 0.288 | 0.219** |
| SVF | -0.027 | -0.005 | -0.001 | -0.001 | 0.003 | -0.006** |
| Land use: funeral | -0.078 | -0.003 | 0.004 | 0.012 | 0.019 | 0.015** |
| Adjusted R-squared: 75.7 %; AIC: 1644.835 | | | | | | |

**p<0.001

a: estimated from simple linear regression

b: estimated from geographically weighted regression
