# Supplementary material for: Exploring the spatial association between the distribution of temperature and urban morphology with green view index
Source: PLoS One. 2024 May 14;19(5):e0301921. doi: 10.1371/journal.pone.0301921 (PMC11093354; doi:10.1371/journal.pone.0301921)

Appendix 4. Geographical distributions of the selected significant predictors of monthly average temperature difference

(A) Green view index

(B) Sky view factors.

(C) Land use: funerals


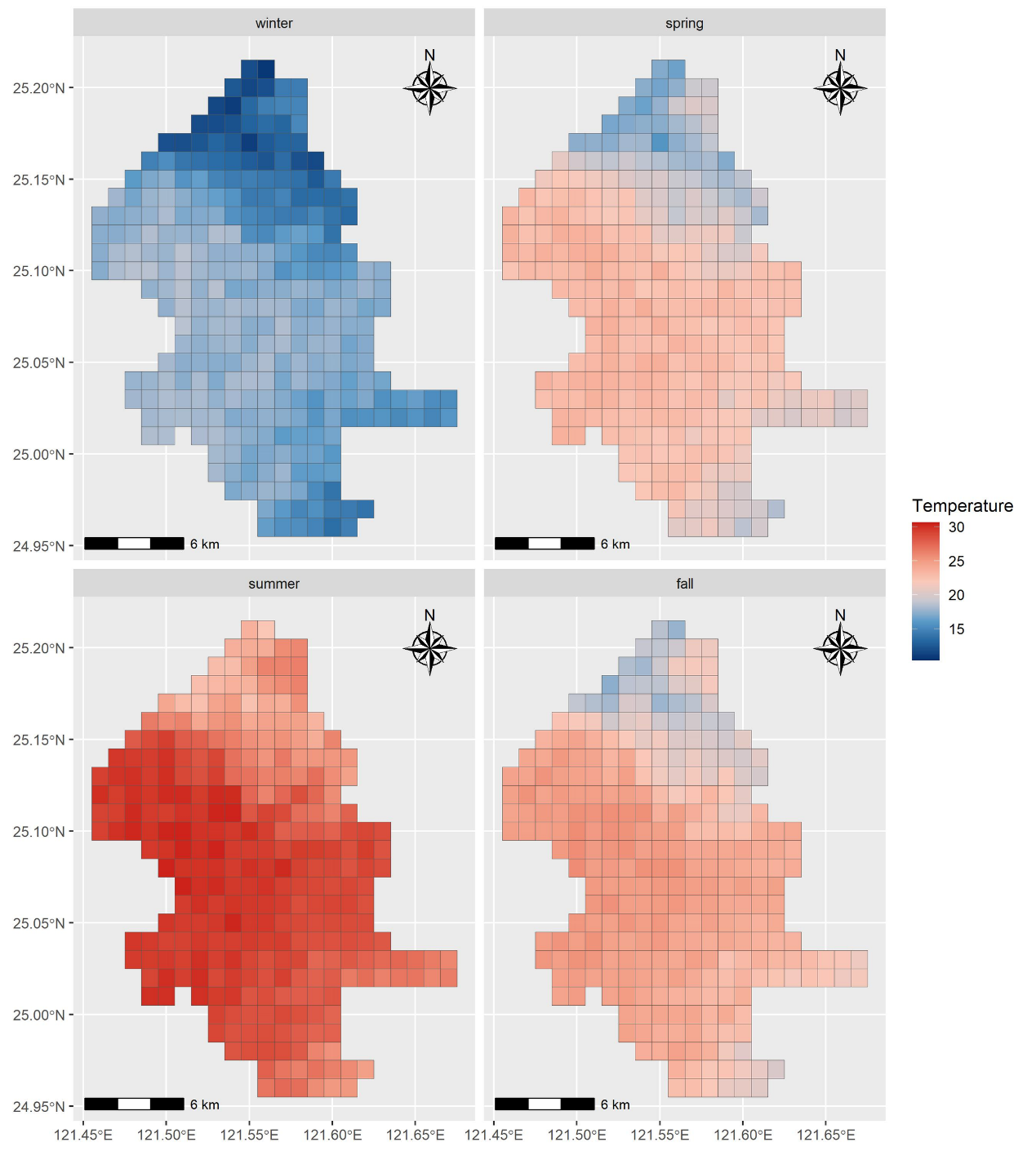

Supplement: S4 Appendix — (DOCX) [file pone.0301921.s004.docx]
