# Supplementary material for: Exploring the spatial association between the distribution of temperature and urban morphology with green view index
Source: PLoS One. 2024 May 14;19(5):e0301921. doi: 10.1371/journal.pone.0301921 (PMC11093354; doi:10.1371/journal.pone.0301921)
Supplement: Attachment — Submitted filename: Response_letter.docx [file pone.0301921.s005.docx]

Journal Requirements:

When submitting your revision, we need you to address these additional requirements.

1. Please ensure that your manuscript meets PLOS ONE's style requirements, including those for file naming. The PLOS ONE style templates can be found at
   <https://journals.plos.org/plosone/s/file?id=wjVg/PLOSOne_formatting_sample_main_body.pdf> and <https://journals.plos.org/plosone/s/file?id=ba62/PLOSOne_formatting_sample_title_authors_affiliations.pdf>

Response: The revised document follows the two style templates.

2. Please note that PLOS ONE has specific guidelines on code sharing for submissions, in which author-generated code underpins the findings in the manuscript. In these cases, all author-generated code must be made available without restrictions upon publication of the work. Please review our guidelines at <https://journals.plos.org/plosone/s/materials-and-software-sharing#loc-sharing-code> and ensure that your code is shared in a way that follows best practice and facilitates reproducibility and reuse.

Response: We have prepared the codes and data used in this study to make them suitable for deposition in the open repository figshare.com.

3. We note that the grant information you provided in the ‘Funding Information’ and ‘Financial Disclosure’ sections do not match.

When you resubmit, please ensure that you provide the correct grant numbers for the awards you received for your study in the ‘Funding Information’ section.

Response: I have checked the submission system and the manuscript. The correct funding information (see below) is provided in the manuscript.

“This study was supported by a grant from the National Science and Technology Council of Taiwan (grant number MOST 111-2121-M-001-002).”

3. We note that you have indicated that data from this study are available upon request. PLOS only allows data to be available upon request if there are legal or ethical restrictions on sharing data publicly. For more information on unacceptable data access restrictions, please see <http://journals.plos.org/plosone/s/data-availability#loc-unacceptable-data-access-restrictions>.

Response: We provide the codes and data that we used, which are provided in the open depository.

“The data and code used in this study have been uploaded to a public depository (https://doi.org/10.6084/m9.figshare.24922275; https://doi.org/10.6084/m9.figshare.24922278).”

In your revised cover letter, please address the following prompts:

a) If there are ethical or legal restrictions on sharing a de-identified data set, please explain them in detail (e.g., data contain potentially sensitive information, data are owned by a third-party organization, etc.) and who has imposed them (e.g., an ethics committee). Please also provide contact information for a data access committee, ethics committee, or other institutional body to which data requests may be sent.

b) If there are no restrictions, please upload the minimal anonymized data set necessary to replicate your study findings as either Supporting Information files or to a stable, public repository and provide us with the relevant URLs, DOIs, or accession numbers. For a list of acceptable repositories, please see <http://journals.plos.org/plosone/s/data-availability#loc-recommended-repositories>.

We will update your Data Availability statement on your behalf to reflect the information you provide.

Response: We have changed our data availability statement to meet your requirements.

“The data and code used in this study have been uploaded to a public depository (<https://doi.org/10.6084/m9.figshare.24922275>; https://doi.org/10.6084/m9.figshare.24922278).”

4. We note that Figure 1 in your submission contain copyrighted images. All PLOS content is published under the Creative Commons Attribution License (CC BY 4.0), which means that the manuscript, images, and Supporting Information files will be freely available online, and any third party is permitted to access, download, copy, distribute, and use these materials in any way, even commercially, with proper attribution. For more information, see our copyright guidelines: <http://journals.plos.org/plosone/s/licenses-and-copyright>.
    
   We require you to either (1) present written permission from the copyright holder to publish these figures specifically under the CC BY 4.0 license, or (2) remove the figures from your submission:
    
   A.) You may seek permission from the original copyright holder of Figure 1 to publish the content specifically under the CC BY 4.0 license. 
    
   We recommend that you contact the original copyright holder with the Content Permission Form (<http://journals.plos.org/plosone/s/file?id=7c09/content-permission-form.pdf>) and the following text:
   “I request permission for the open-access journal PLOS ONE to publish XXX under the Creative Commons Attribution License (CCAL) CC BY 4.0 (<http://creativecommons.org/licenses/by/4.0/>). Please be aware that this license allows unrestricted use and distribution, even commercially, by third parties. Please reply and provide explicit written permission to publish XXX under a CC BY license and complete the attached form.”
    
   Please upload the completed Content Permission Form or other proof of granted permissions as an ""Other"" file with your submission. 
    
   In the figure caption of the copyrighted figure, please include the following text: “Reprinted from [ref] under a CC BY license, with permission from [name of publisher], original copyright [original copyright year].”

Response: We have checked the Google Street View license (<https://about.google/brand-resource-center/products-and-services/geo-guidelines/#street-view>). They do not allow the publication of street-view imagery. Thus, we have removed the original Fig. 1 and Fig. 2.

B.) If you are unable to obtain permission from the original copyright holder to publish these figures under the CC BY 4.0 license or if the copyright holder’s requirements are incompatible with the CC BY 4.0 license, please either i) remove the figure or ii) supply a replacement figure that complies with the CC BY 4.0 license. Please check copyright information on all replacement figures and update the figure caption with source information. If applicable, please specify in the figure caption text when a figure is similar but not identical to the original image and is therefore for illustrative purposes only.

5. We note that Figure 3 - 7 and Appendix 1 in your submission contain map images which may be copyrighted. All PLOS content is published under the Creative Commons Attribution License (CC BY 4.0), which means that the manuscript, images, and Supporting Information files will be freely available online, and any third party is permitted to access, download, copy, distribute, and use these materials in any way, even commercially, with proper attribution. For these reasons, we cannot publish previously copyrighted maps or satellite images created using proprietary data, such as Google software (Google Maps, Street View, and Earth). For more information, see our copyright guidelines: <http://journals.plos.org/plosone/s/licenses-and-copyright>.
    
   We require you to either (1) present written permission from the copyright holder to publish these figures specifically under the CC BY 4.0 license, or (2) remove the figures from your submission:
    
   A.) You may seek permission from the original copyright holder of Figure 3 - 7 and Appendix 1 to publish the content specifically under the CC BY 4.0 license.

Response: The original Figures 3–7 have now become Figures 1–5. The base map is from OpenStreetMap (openstreetmap.org), which is under the license of CC BY 4.0. The authors computed and created the overlay layer. We have added the source of the base map to the figure captions.

Reviewers' comments:

Review Comments to the Author
Reviewer #1: The article considers an important research subject. However, the data presented is overloaded information, which needs simplification. There are some questions, which needs to be considered for clarification/explanation of the methods. A few examples are below:

Introduction" Lines 59 - 61: What type of discomfort? Need explanation?

Response: Thank you for your valuable comments. We have modified the text to make the point clearer. (Lines 49–52 in revised version).

“Green spaces or green planting measures act as regulators, helping to mitigate the urban heat island effect [3, 4]. Furthermore, a lack of green spaces affects people's well-being and mental health and can even increase the risk of mortality [5, 6]. “

Lines 62-65: This is not clear. Needs rephrasing and further clarification. How the two are indicators? What these indicate to. Instead, both are methods and can be applied for deriving indicators or relationships?

Response: Thank you. We have revised the text to provide greater clarity. (Lines 52–59).

“Satellite image-derived metrics [5], such as the Normalized Difference Vegetation Index (NDVI) and Enhanced Vegetation Index (EVI), which quantify greenness at the grid level, and geographic information systems, used to calculate the spatial accessibility of park green spaces at the community level [7], are popular ways of computing proxy indicators of urban green space. Satellite imagery offers continuous spatial coverage of greening data, highly correlated with the spatial accessibility of parks or other open green spaces in a community.”

Line 65: Spatial or temporal resolution?

Response: Thank you. We have added “spatial” to the sentence. (Lines 59–61)

“However, satellite images must be taken from above, resulting in limitations in spatial resolution and shooting angles. Tight building layouts may obscure ground-level images and vertical views of buildings.”

Line 65: Shooting angle limitations: Would factors like shadows of tall buildings and planning be limiting factors?

Response: Thank you. We have rewritten this sentence to explain the concept better. (Lines 59–61)

“However, satellite images must be taken from above, resulting in limitations in spatial resolution and shooting angles. Tight building layouts may obscure ground-level images and vertical views of buildings.”

Line 70: What is meant by thermal comfort?

Response: Thanks. We have added an explanation. (Lines 63–67)

“In cities, in addition to large parks and green spaces, street trees or plants cultivated by residents are closer to their daily living environments and may directly contribute to their thermal comfort, namely, the subjective evaluation of the thermal environment, noise relief, and air purification.”

Line 84: replace "studied" with "recognized" or "admitted" or "considered" or "presented"

Response: Thank you. We have repharased the sentence.

Line 89: Which environmental factors?

Response: We have listed the environmental factors in the sentence. (Lines 82–86).

“Unlike previous studies that focused on urban parks or satellite imagery, this study incorporated new ecological indicators, including GVI, building view factor (BVF), and Sky View Factor (SVF), calculated from GSV and other environmental data, including satellite images, altitude, population, building areas, traffic volume, and land-use information.”

Line 97: Parameters instead of indicators?

Response: Thanks. We believe that indicators are the more appropriate term. These indicators were developed to quantify the greenness, openness of sky views, and building density.

Definition and computation of GVI 2.0
Line 120: How is this different than the first generation. The first generation GVI used six pictures with angles 0 to 180? The difference of splitting a picture to two is not what should be a version difference? Using pixels ultimately is spatial coverage or area, so is not different than using area, which was in the first generation or the referenced modified GVI?

Response: We apologize for the confusion regarding the definition between GVI 1.0 and GVI 2.0. The main difference is the algorithm used to identify the pixels in green space. Segmentation at different angles is only related to the number of calculated files. We revised the sentence to clarify this. We have added this sentence to the revised manuscript. (Lines 123–125).

“The main difference between GVI 1.0, and GV 2.0, is that the GVI 2.0 algorithm improves the accuracy of tree cover identification by learning from many publicly available labeled street images.”

Normalized Difference Vegetation Index (NDVI)
Line 157: What is meant by bird's eye satellite images?

Response: Satellite images were taken from directly overhead, like a bird’s-eye view, and showed the scene on the ground. I mentioned this in an earlier paragraph. I have deleted it here for conciseness.

Line 162: With the limiting factors described in the introduction section, was this spatial resolution efficient to capture greenness in an urban environment? How this could be effective, given high rise building covering a street view from the sky at some angle of view?

Response: Thank you. Satellite images used to identify green spaces in urban areas are affected by tall buildings. However, satellite imagery captures greenness very well in an open space such as a forest or field. We have added this point to the Introduction section.

Land use data
Line 186: The official classification is 9, 48, and 93. How was this classification made to 09 classes and what was the basis for that?

Response: We have added the following statement in the revised text. (Lines 188–192)

“Official land-use classifications are based on the law and previous land-use surveys. Each upper-level classification has different subcategories. The first-tier category is broad and includes agriculture, forestry, transportation, water conservation, construction, public utilities, entertainment, and mineral salt utilization.”

Altitude and the size of the building area
Line 209: How was this integrated with the other spatial resolution datasets, such as the 250 m MODIS or 30m Landsat? Or it was used independent of those? If used independently, then how was this correlated to the GVI calculated from the GSV?

Response: Thank you. In the first sentence of the first paragraph in the section headed “Statistical Model,” we stated that all explanatory data were converted into grid cells to fit our dependent variable, the TCCIP grid. (Line 223–225)

Results:
Line 264: How was this seasonal variation used? It needs to be explained in the methods and that how this impacted the study/methods?

Response: Thank you. We have added the following sentence to the section on the Statistical model. (Lines 236–238).

“Additionally, considering the GVI, we treated the seasons as categorical variables to account for their effect on either the average temperature or the temperature difference.”

Line 322: Is it graveyards or different?

Response: Funeral land in Taiwan can be used for graveyards and bone towers.

Line 323: Graveyards are generally kept greener, a traditional or religious norm across the world. A positive relationship with temperature is considerable.

Response: This is not always the case, as the density of tombstones is usually high, and the entire cemetery is often completely devoid of shade. The image below shows a snapshot of a Taipei City cemetery obtained from Google Satellite Imagery.

Reviewer #2: The authors mentioned the effect of small plant using google street view; however, this kind of analysis requires very high-resolution data. It should be mentioned how this data was acquired and preprocessed before feeding it to the deep learning models.

Response: Thank you for the comment. In the first paragraph of the Methods section, we describe the collection and preprocessing of Google Street View data. We sampled points from the road network at intervals of 30 m, used the Google Map API to retrieve the two pitch angles of the panoramic image, further divided it into six images at different angles, and input them into the deep learning model. Although the resolution is low, the algorithm can identify it well. However, the true greenness value is underestimated if an image is obscured by passing vehicles in certain shooting directions. We have added this information to the Limitations section. (Lines 108–116).

**“Google Street View (GSV) data collection**

We first sampled GSV points at intervals of 30 m from the commercial road network data in Taipei City, Taiwan. We then applied the Google Map API with an authentication code to retrieve our sample points' latest panorama images from 2018 to 2022 (<https://developers.google.com/maps/documentation/streetview/overview>).

We split the panoramic image into six separate images for each sample point, with pitch angles of 0° and 45°. Thus, 12 images were obtained for further GVI computation at each point. The resolution of each image is 224 × 224 pixels. A total of 86,637 sample points and 1,039,644 images were used in this study.“

Limitations

“The third limitation is an inherent feature of GSV. Passing vehicles or other obstacles in certain shooting directions obscure the image, leading to an underestimation of the true greenness value.”(Lines 338–440)

The monthly temperature data that the authors has used is very coarse resolution. How will this resolution affect the study, since the authors are talking about ambient temperatures?

Response: Thank you for the comment. We have added this point under “Limitations” in the revised manuscript. (Lines 440–445)

“The fourth limitation is the temporal discontinuity of the green space indicators. The temperatures for each month represent the average stable conditions for that month. Our primary focus is green space, which does not change much at the daily level but may vary at the monthly level. As a result, we used monthly temperatures as modeling targets since green space indicators cannot be updated frequently.”

In line 61 the authors mentioned that Satellite data such as NDVI. This is not correct. NDVI and EVI are satellite data derived metrics. The whole statement on line 61 needs to be reevaluated and rewritten.

Response: Thank you for the comments. We have corrected the terms. (Lines 52–57)

“Satellite image-derived metrics [5], such as the Normalized Difference Vegetation Index (NDVI) and Enhanced Vegetation Index (EVI), which quantify greenness at the grid level, and geographic information systems, used to calculate the spatial accessibility of park green spaces at the community level [7], are popular ways of computing proxy indicators of urban green space.”

On line 65 what does shooting angle limitations means?

Response: Thank you. Moreover, another reviewer was confused by this term. We have corrected this sentence accordingly. (Lines 59–61).

“However, satellite images must be taken from above, resulting in limitations in spatial resolution and shooting angles. Tight building layouts may obscure ground-level images and vertical views of buildings.”

I think the introduction section needs to be rewritten with a proper background of the study and identifying how this is a gap and how it can be addressed using the proposed study

Response: Thank you for the comment. We have revised the Introduction and tried to improve it in the process.

The methods section is okay. Some more details on how the data was preprocessed before giving it to the model should be added.

Response: Thank you for the comment. We have described more fully how we processed the data.

The discussion needs improvements. The authors can put the results in a proper context and show why this is important and how their results added to the current methods that are being uses.

Response: Thank you for the comment. We have revised the Discussion and tried to improve it in the process.

I would suggest the authors to make the changes and also revise the sentence structure and grammatical issues.

Response: Thank you for the comment. We have considered your points, rewritten the sentences, and corrected the grammatical issues.
